# Determining the Predominant Lesion in Patients With Severe Aortic Stenosis and Coronary Stenoses: A Multicenter Study Using Intracoronary Pressure and Flow

**DOI:** 10.1161/CIRCINTERVENTIONS.119.008263

**Published:** 2019-11-22

**Authors:** Yousif Ahmad, Jeroen Vendrik, Ashkan Eftekhari, James P. Howard, Christopher Cook, Christopher Rajkumar, Iqbal Malik, Ghada Mikhail, Neil Ruparelia, Nearchos Hadjiloizou, Sukhjinder Nijjer, Rasha Al-Lamee, Ricardo Petraco, Takayuki Warisawa, Gilbert W.M. Wijntjens, Karel T. Koch, Tim van de Hoef, Guus de Waard, Mauro Echavarria-Pinto, Angela Frame, Nilesh Sutaria, Gajen Kanaganayagam, Ben Ariff, Jon Anderson, Andrew Chukwuemeka, Michael Fertleman, Sasha Koul, Juan F. Iglesias, Darrel Francis, Jamil Mayet, Patrick Serruys, Justin Davies, Javier Escaned, Niels van Royen, Matthias Götberg, Christian Juhl Terkelsen, Evald Høj Christiansen, Jan J. Piek, Jan Baan, Sayan Sen

**Affiliations:** 1 National Heart and Lung Institute, Hammersmith Hospital, Imperial College London, United Kingdom (Y.A., J.P.H., C.C., C.R., R.A.-L., R.P., T.W., D.F., J.M., P.S., S.S.).; 2 Amsterdam UMC, University of Amsterdam, Heart Center, Department of Clinical and Experimental Cardiology, the Netherlands (J.V., K.T.K., T.v.d.H., J.J.P., J.B.).; 3 Aarhus University Hospital Skejby, Denmark (A.E., C.J.T., E.H.C.).; 4 Department of Cardiology, Hammersmith Hospital, Imperial College Healthcare NHS Trust, London, United Kingdom (I.M., G.M., N.R., N.H., S.N., A.F., N.S., G.K., B.A., J.A., A.C., M.F., J.D.).; 5 Department of Cardiology, VU University Medical Center, Amsterdam, the Netherlands (G.d.W., N.v.R.).; 6 Hospital Clínico San Carlos, Madrid, Spain (M.E.-P., J.E.).; 7 Department of Cardiology, Clinical Sciences, Lund University, Skåne University Hospital, Sweden (S.K., M.G.).; 8 Cardiology Department, Lausanne University Hospital, Switzerland (J.F.I.).

**Keywords:** aortic valve stenosis, diastole, hyperemia, microcirculation, myocardium

## Abstract

Patients with severe aortic stenosis (AS) often have coronary artery disease. Both the aortic valve and the coronary disease influence the blood flow to the myocardium and its ability to respond to stress; leading to exertional symptoms. In this study, we aim to quantify the effect of severe AS on the coronary microcirculation and determine if this is influenced by any concomitant coronary disease. We then compare this to the effect of coronary stenoses on the coronary microcirculation.

**Methods:**

Group 1: 55 patients with severe AS and intermediate coronary stenoses treated with transcatheter aortic valve implantation (TAVI) were included. Group 2: 85 patients with intermediate coronary stenoses and no AS treated with percutaneous coronary intervention were included. Coronary pressure and flow were measured at rest and during hyperemia in both groups, before and after TAVI (group 1) and before and after percutaneous coronary intervention (group 2).

**Results:**

Microvascular resistance over the wave-free period of diastole increased significantly post-TAVI (pre-TAVI, 2.71±1.4 mm Hg·cm·s^−1^ versus post-TAVI 3.04±1.6 mm Hg·cm·s^−1^ [*P*=0.03]). Microvascular reserve over the wave-free period of diastole significantly improved post-TAVI (pre-TAVI 1.88±1.0 versus post-TAVI 2.09±0.8 [*P*=0.003]); this was independent of the severity of the underlying coronary stenosis. The change in microvascular resistance post-TAVI was equivalent to that produced by stenting a coronary lesion with an instantaneous wave-free ratio of ≤0.74.

**Conclusions:**

TAVI improves microcirculatory function regardless of the severity of underlying coronary disease. TAVI for severe AS produces a coronary hemodynamic improvement equivalent to the hemodynamic benefit of stenting coronary stenoses with instantaneous wave-free ratio values <0.74. Future trials of physiology-guided revascularization in severe AS may consider using this value to guide treatment of concomitant coronary artery disease.

**WHAT IS KNOWN**

- Patients with severe aortic stenosis undergoing transcatheter aortic valve implantation often have concomitant coronary artery disease. If they present with chest pain or dyspnea, it can be unclear if the coronary stenosis or the aortic valve stenosis is responsible for an individual patient’s symptoms.
- It has been demonstrated that hyperemic indices are significantly affected by severe aortic stenosis, with hyperemic flow increasing significantly after transcatheter aortic valve implantation. Resting hemodynamics are less susceptible to this, specifically in the wave-free period of diastole, where it has previously been shown that flow during this period is unchanged post-transcatheter aortic valve implantation.

**WHAT THE STUDY ADDS**

- Transcatheter aortic valve implantation improves microcirculatory function regardless of the severity of underlying coronary disease.
- This improvement in microcirculatory function is only matched by stenting coronary lesions with an instantaneous wave-free ratio <0.74.
- The predominant lesion affecting microvascular resistance in patients with severe aortic stenosis and coronary stenoses seems to be the aortic stenosis unless the instantaneous wave-free ratio value is ≤0.74.

Patients with severe aortic stenosis (AS) often have coronary artery disease (CAD).^1^ If they present with chest pain or dyspnea, it can be unclear if the coronary stenosis or the aortic valve stenosis is responsible for an individual patient’s symptoms.

The cause of chest pain or shortness of breath in these patients is a result of an inability of the microcirculation to increase blood flow in response to increased demand. While the effects of both the coronary lesion^2^ and the aortic valvular stenosis^3^ on the microcirculation have been individually studied, it is not known how the 2 interact when they are present in the same patient, and which is the predominant lesion.

It has been demonstrated that hyperemic indices are significantly affected by severe AS,^4^ with hyperemic flow increasing significantly after transcatheter aortic valve implantation (TAVI). Resting hemodynamics are less susceptible to this, specifically in the wave-free period of diastole, where it has previously been shown that flow during this period is unchanged post-TAVI.

This study uses the resting hemodynamics of the wave-free period to determine the relative effects of TAVI and percutaneous coronary intervention (PCI) on myocardial perfusion. Microvascular resistance over the wave-free period has been demonstrated to reflect coronary stenosis severity^5^; with low resistance suggesting a more severe stenosis and higher resistance suggesting a less severe stenosis. It has also been shown that microvascular resistance over the wave-free period is affected by severe AS. In patients with both severe AS and CAD, the relative contribution of each to microvascular resistance is unknown.

In this study, we aim to determine when a patient has severe AS and coronary disease which is the predominant lesion affecting myocardial blood flow. We aim to do this by (1) quantifying the effect of severe AS on the function of the coronary microcirculation and determine if this is influenced by concomitant coronary disease; (2) quantifying the effect of a coronary stenosis on the function of the coronary microcirculation; and (3) determining the severity of coronary stenosis that, when stented, provides equivalent improvement in microcirculatory function as TAVI.

## Methods

The methods and materials that support the findings of this study are available from the corresponding author on reasonable request.

### Patient Population

Part 1: 55 patients with severe AS undergoing TAVI with moderate coronary lesions were recruited from 4 European centres (The Hammersmith Hospital, Imperial College Healthcare NHS Trust, London, United Kingdom; Amsterdam Medical Center, Amsterdam, the Netherlands; Skane University Hospital, Lund, Sweden; and Aarhus University Hospital, Aarhus, Denmark). The study was approved by an institutional review committee at each site. All patients had prospectively collected combined coronary pressure and flow measurements, with paired measurements pre- and post-TAVI. All patients were scheduled for TAVI on clinical grounds in accordance with clinical guidelines,^6^ after a decision at a Heart Team meeting, and gave written informed consent for the study protocol. Exclusion criteria were known nonviable myocardium in the area of the corresponding coronary artery being studied, contra-indication to the administration of adenosine, inability to consent or weight over 200 kg. All patients had concomitant AS and CAD, and physiological assessments were performed in each patient. There was no PCI performed in this group, after Heart Team decision that it was not required before TAVI and enrollment in the study.

Part 2: 85 patients with intermediate coronary lesions undergoing PCI were included from 4 European centres as part of the IDEAL collaboration^2^ (Amsterdam Medical Center, Amsterdam, the Netherlands; The Hammersmith Hospital, Imperial College Healthcare NHS Trust, London, United Kingdom; Hospital Clinico San Carlos, Madrid, Spain; and VU University Medical Center, Amsterdam, the Netherlands). The study was approved by an institutional review committee at each site. All patients had combined coronary pressure and flow measurements, with paired measurements pre- and post-PCI. All patients recruited were scheduled for elective coronary angiography with physiological stenosis assessment by fractional flow reserve and gave written informed consent for acquisition of additional physiological data for study purposes. Exclusion criteria were acute myocardial infarction within 48 hours; contraindication to the administration of adenosine; severe valvular heart disease; weight >200 kg; previous coronary artery bypass surgery; vessels with angiographically identifiable myocardial bridging or collateral arteries; and vessels supplying an infarcted territory. All patients in this group underwent PCI as had been determined by the treating clinical team.

### Cardiac Catheterization Protocol

In all patients, cardiac catheterization and coronary angiography was performed via either the transradial or transfemoral route at the operator’s discretion. A guiding catheter was used to intubate the target artery. Therapeutic dose heparin was administered. A dual pressure and Doppler sensor-equipped 0.014” guidewire was used for all physiological assessments (ComboWire, Volcano Corp, San Diego, CA). The pressure signals were normalized in the aorta before advancing the wire a minimum of 3-vessel diameters distal to the coronary stenosis. Doppler signals were optimized and stabilized to ensure good tracking profiles. All flow measurements were made by experienced operators; the reproducibility of flow measurements in such hands has been previously demonstrated.^7^ At this stage, resting pressure and flow measurements were recorded. Hyperemia was then induced using adenosine, either as an intracoronary bolus of 150 μg or an intravenous infusion of 140 μg/kg per minute. Physiological measurements under hyperemic conditions were then recorded. At the end of each recording, the pressure sensor was returned to the catheter tip to ensure that there was no pressure drift. When drift was identified (≥0.02), all measurements were repeated. For TAVI patients, left ventricular pressures were recorded using a pigtail catheter placed in the LV cavity. All patients then either underwent PCI (for patients without AS) or TAVI (for patients with AS). Subsequent to either intervention, the entire protocol was repeated with the wire sited in the same location as the preintervention measurements.

### PCI Procedures

Drug-eluting stents were used as standard of care. Optimization using intracoronary imaging and postdilatation were performed at the operator’s discretion.

### TAVI Procedures

All patients were treated under local anesthesia and conscious sedation. The valves used were either the Edward’s Sapien XT/S3 valves (Edwards Lifesciences LCC, Irvine, CA), the Medtronic CoreValve/Evolut-R valves (Medtronic, Inc, Minneapolis, MN), or Lotus valve (Boston Scientific, Natick, MA). Valve choice was at the Heart Team and operator’s discretion.

### Analysis of Hemodynamic Data

ECG, pressure, and flow velocity signals were processed with the dedicated device console (ComboMap; Volcano Corp, San Diego, CA). Analog output feeds were taken from the pressure-velocity console and ECG, fed into a National Instruments DAQ-Card AI-16E-4, and acquired at 1 kHz with Labview. Data were analyzed offline with a custom software package designed with Matlab (Mathworks, Natick, MA), which permitted phasic analysis including that of the wave-free period. The wave-free period was identified using wave-intensity analysis^7^ and used to perform phasic analysis. Coronary pressure, flow, and resistance were measured during resting conditions and during hyperemia.

Microvascular reserve was derived as a metric of improvement in coronary hemodynamics after intervention. This was defined as a ratio of hyperemic microvascular resistance to resting microvascular resistance.

Definitions of other hemodynamic variables were as follows:


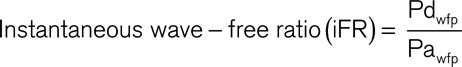



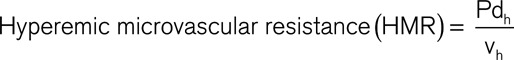



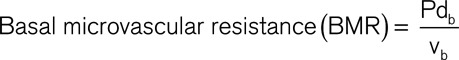



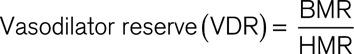



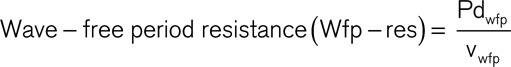


Where Pa=mean aortic pressure; Pd=mean intracoronary pressure distal to a stenosis; Wfp=the wave-free period of diastole; v_h_=mean flow velocity distal to a stenosis during hyperemia; v_b_=mean flow velocity distal to a stenosis at baseline.

## Statistical Methods

Continuous variables are presented as mean and SD unless otherwise stated. Comparisons before and after intervention were performed with a paired *t* test for continuous variables. Paired ordinal categorical data such as LV function were analyzed using the Wilcoxon signed-rank test. The threshold for statistical significance was set at 0.05. Pearson correlation coefficient was used to assess correlations. All analyses were performed using R version 3.2.1 (R Foundation for Statistical Computing, Vienna, Austria).

## Results

### Patient Population

#### TAVI Group

Fifty-five patients (81.7 [±5.9] years; 49.1% male) were included. Baseline clinical characteristics are shown in Table 1. The baseline echocardiographic characteristics are summarized in Table 2.

**Table 1.**
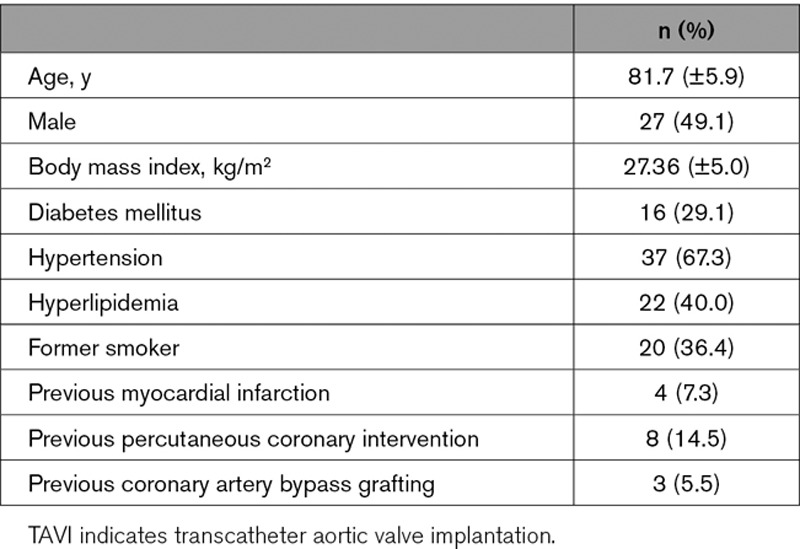
Baseline Clinical Characteristics of TAVI Group

**Table 2.**
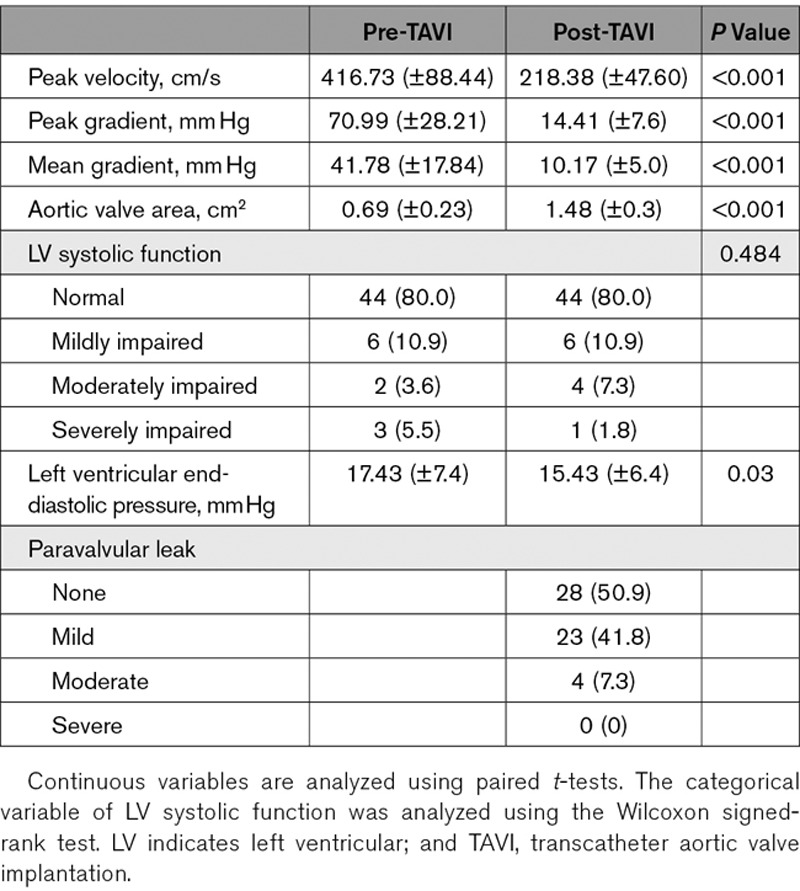
Baseline Echocardiographic Characteristics of TAVI Group

#### PCI Group

Eighty-five patients were included (61.3 [±9.4] years; 74.1% male). Baseline clinical characteristics are shown in Table 3.

**Table 3.**
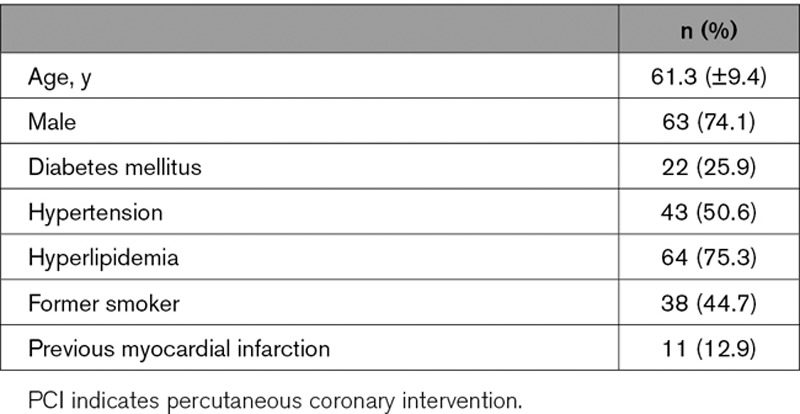
Baseline Clinical Characteristics of PCI Group

### Quantitative Coronary Angiography

#### TAVI Group

Quantitative coronary angiography for the patients undergoing TAVI is summarized in Table 4.

**Table 4.**
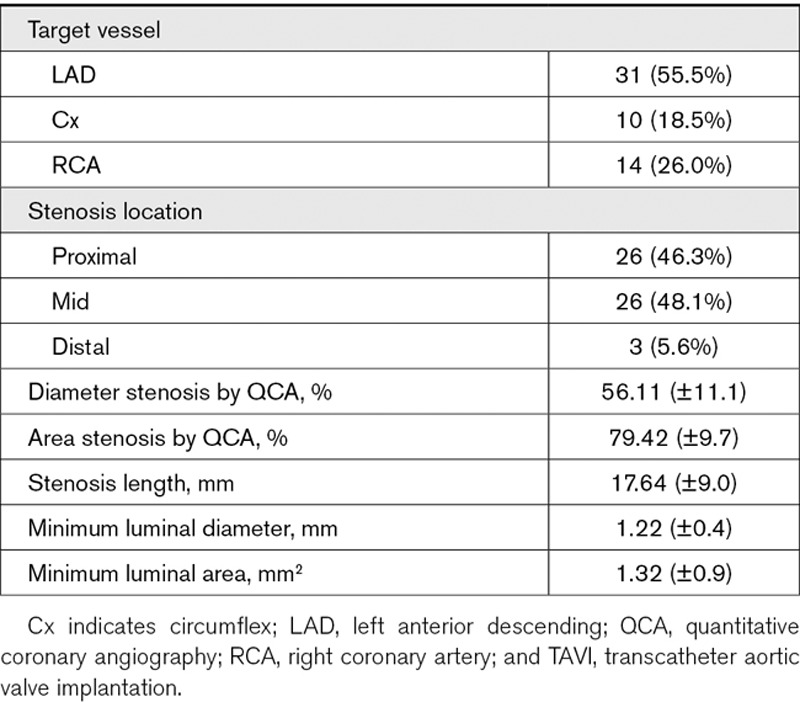
Quantitative Coronary Angiographic Data for TAVI Patients

#### PCI Group

Quantitative coronary angiography for the patients undergoing PCI is summarized in Table 5.

**Table 5.**
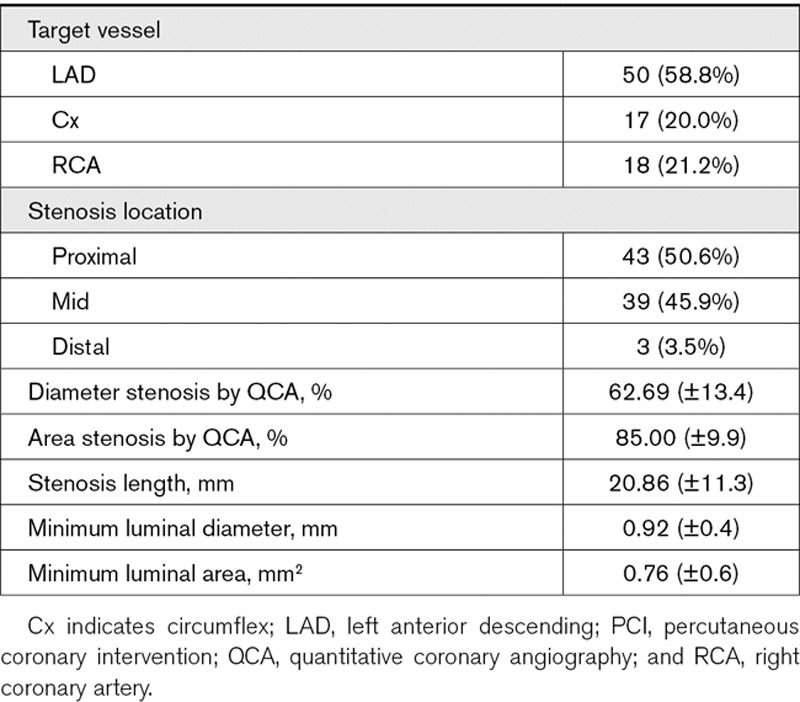
Quantitative Coronary Angiographic Data for PCI Patients

### Coronary Physiological Parameters Before and After TAVI

Commonly reported coronary physiological parameters before and after TAVI are summarized in Table 6. There was a significant reduction in fractional flow reserve immediately (*P*<0.001) post-TAVI, and a significant increase in coronary flow reserve immediately post-TAVI (*P*=0.03). Instantaneous wave–free ratio (iFR) was unchanged immediately post-TAVI (*P*=0.80).

**Table 6.**
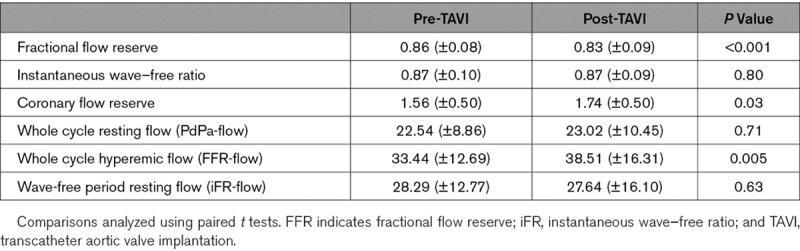
Values of Common Coronary Physiological Indices Pre- and Post-TAVI

### Microvascular Resistance Over the Wave-Free Period of Diastole Before and After TAVI

**Figure 1.**
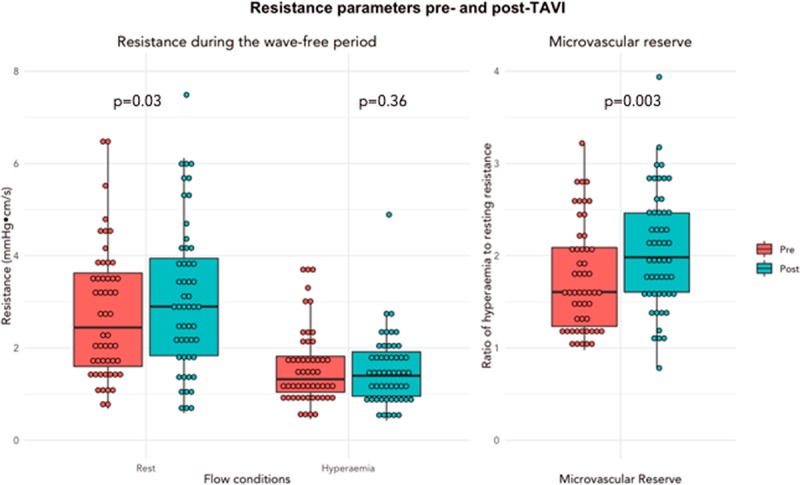
**Figure outlining the changes in resting resistance, hyperemic resistance, and microvascular reserve pre- and post-transcatheter aortic valve implantation (TAVI).**

Changes in resistance after TAVI are summarized in Figure 1. Resting resistance over the wave-free period of diastole increased significantly post-TAVI (pre-TAVI 2.71±1.4 mm Hg·cm·s^−1^ versus post-TAVI 3.04±1.6 mm Hg·cm·s^−1^ [*P*=0.03]). Hyperemic resistance over the wave-free period of diastole did not change post-TAVI (pre-TAVI 1.58±0.8 mm Hg·cm·s^−1^ versus post-TAVI 1.49±0.7 mm Hg·cm·s^−1^ [*P*=0.36]). Microvascular reserve over the wave-free period of diastole significantly improved post-TAVI (pre-TAVI 1.88±1.0 versus post-TAVI 2.09±0.8 [P=0.003]).

### Improvement in Wave-Free Period Resistance Achieved by TAVI and PCI According to Baseline iFR

**Figure 2.**
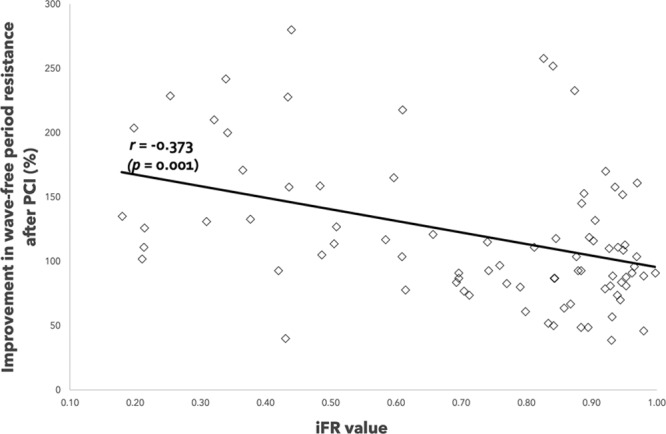
**Correlation between underlying coronary stenosis severity (baseline instantaneous wave–free ratio [iFR] value) and improvement in resistance after percutaneous coronary intervention (PCI) with a strongly statistically significant association.**

Overall, microvascular resistance over the wave-free period improved significantly post-PCI. This improvement was dependent on the baseline iFR value (Figure 2). The more severe the coronary stenosis, the greater the improvement in microvascular resistance. Therefore, microvascular resistance over the wave-free period is a marker of coronary stenosis severity.

**Figure 3.**
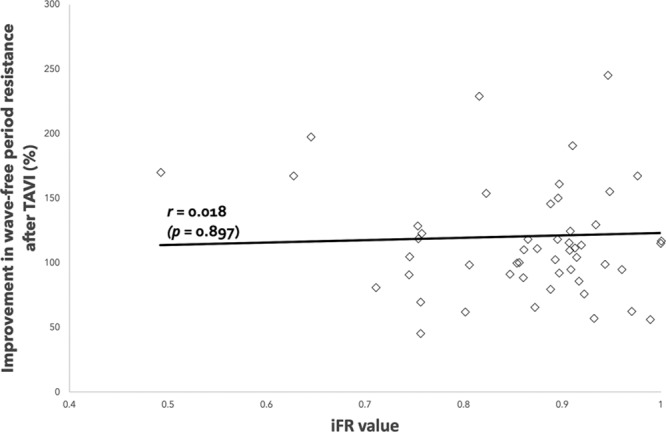
**Correlation between underlying coronary stenosis severity (baseline instantaneous wave–free ratio [iFR] value) and improvement in resistance after transcatheter aortic valve implantation (TAVI) with no significant association seen.**

Overall, microvascular resistance over the wave-free period improved significantly post-TAVI (Figure 3). This improvement was independent of the baseline iFR value: the improvement in microvascular resistance over the wave-free period post-TAVI is consistent across the spectrum of coronary stenosis severity.

### Comparison of the Change in Microvascular Resistance Observed After TAVI and PCI

**Figure 4.**
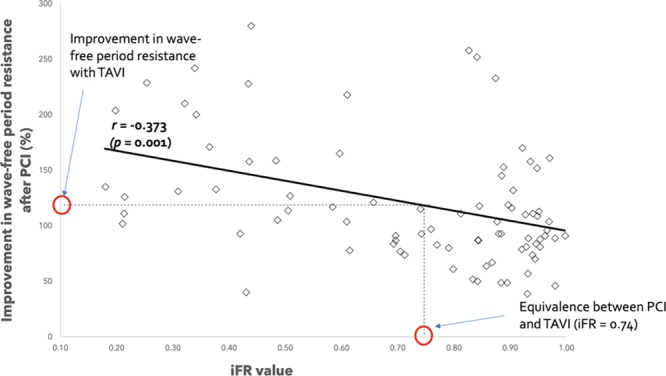
**Correlation between underlying coronary stenosis severity (baseline instantaneous wave–free ratio [iFR] value) and improvement in resistance after percutaneous coronary intervention (PCI**). When the improvement in resistance achieved with transcatheter aortic valve implantation (TAVI) is interpolated, equipoise between PCI and TAVI is seen at iFR values of 0.74.

The average improvement in microvascular resistance over the wave-free period post-TAVI was 19.2±0.5%. Interpolating this data to the improvement of microvascular resistance over the wave-free period post-PCI suggests that at the iFR value 0.74, there is equipoise in the improvement achieved with PCI and TAVI (see Figure 4). That is, stenting lesions with iFR values of 0.74 also provides a 19% improvement in resistance (the same as TAVI). Therefore, the improvement in resistance achieved by PCI seems only able to surpass TAVI when the baseline iFR is <0.74.

## Discussion

In this study, we have shown that (1) in patients with severe AS and intermediate coronary lesions, treatment of the valve results in a significant increase in microvascular resistance; (2) this increase is independent of the severity of the underlying coronary lesion; and (3) TAVI for severe AS produces a hemodynamic improvement equivalent to the hemodynamic benefit of stenting coronary stenoses with iFR values <0.74.

### Microvascular Reserve and Coronary Stenoses

**Figure 5.**
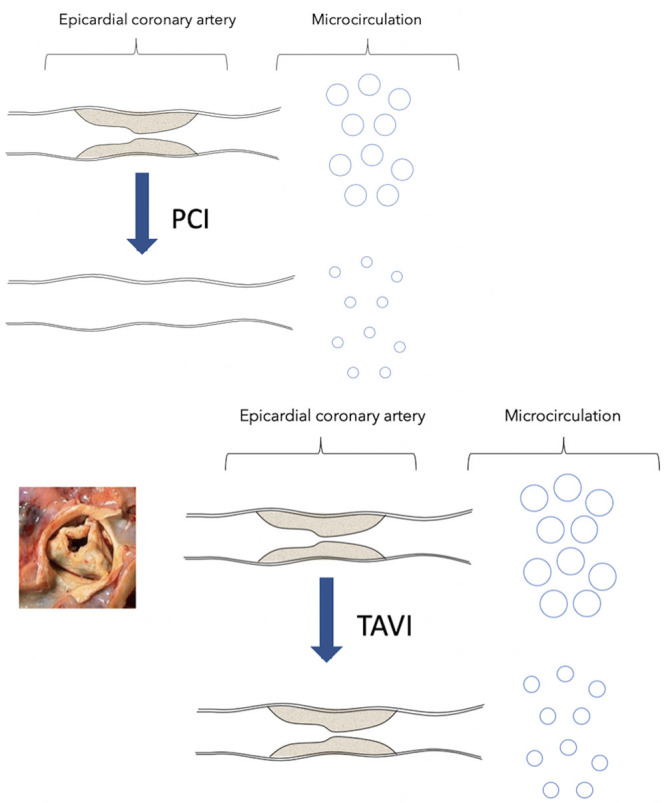
**Figure outlining coronary autoregulation in patients with coronary stenoses and aortic stenosis**. In the top, a patient with a severe coronary stenosis; here, the microcirculation is relatively dilated at rest to maintain coronary flow. In these patients, when the need arises to increase coronary flow further, the capacity of the microcirculation to dilate further to increase flow is limited; therefore, the difference between resting and hyperemic flow (microvascular reserve) is small. In patients with no coronary stenosis, or after percutaneous coronary intervention (PCI), the opposite is true. In these patients, the microcirculation is relatively constricted. Therefore, when the need arises to increase coronary flow further, the capacity of the microcirculation to dilate further to increase flow is large; and the difference between resting and hyperemic flow (microvascular reserve) is also large, resulting in greater microvascular reserve. In the bottom, a patient with coronary stenosis and aortic stenosis. As the aortic valve stenoses, so the microcirculation dilates to maintain coronary flow and microvascular reserve is depleted. Therefore, in patients with severe aortic stenosis and coronary disease, the microcirculation is adapting to 2 variables that affect blood flow: the stenosed aortic valve and the stenosis in the coronary artery. Post-transcatheter aortic valve implantation (TAVI), resting microvascular resistance increases because one lesion affecting coronary flow has been treated. It does not normalize, however, as there is a residual coronary stenosis that needs to be accommodated.

Microvascular reserve reflects the ability of the microcirculation to increase the blood supply to the heart in response to increased demand or workload. In patients with coronary disease, this ability to respond to increased work load is related to (1) the severity of the stenosis within the epicardial artery^2^ and (2) autoregulation of coronary blood flow^8^ and its effect on microvascular resistance. In patients with severe coronary disease, microvascular resistance is relatively lower than in patients with no coronary stenosis (Figure 5).

### AS and Coronary Stenosis

In this study, we demonstrate that AS also influences coronary microvascular tone and its ability to respond to stress. In patients with AS, microvascular resistance at rest is significantly lower than that of post-TAVI patients. This suggests that the coronary microcirculation treats AS similarly to a coronary stenosis. As the aortic valve becomes more and more constricted, the microcirculation dilates to maintain coronary flow and microvascular reserve is depleted (Figure 5).

Therefore, in patients with severe AS and coronary disease, the microcirculation is adapting to 2 variables that affect blood flow: the stenosed aortic valve and the stenosis in the coronary artery. Resting microvascular resistance and therefore microvascular reserve in these patients is therefore limited by both.

Because the microcirculation treats the 2 stenoses similarly, determining the predominant lesion is akin to attempting to determine which stenosis is predominant in a vessel with tandem lesions. It has been demonstrated that this is not possible with hyperemia due to both stenoses influencing the blood flow across the other.^9^ However, it has been demonstrated to be possible to isolate the significance of a specific lesion with iFR.^10^

This finding for tandem lesions can be extrapolated to the TAVI population. In these patients, the tandem lesions are the coronary stenosis and the aortic valvular stenosis. Placing a pressure wire in the vessel distal to the coronary stenosis in a patient with severe AS is therefore analogous to placing a pressure wire between 2 serial lesions in a coronary artery. We have previously demonstrated that hyperemic flow changes significantly post-TAVI suggesting that hyperemic indices cannot be used to isolate coronary stenosis severity in the context of severe AS. However, we have demonstrated that iFR can accurately isolate the coronary stenosis severity independent to the aortic valve in this setting^4^; the iFR pre-TAVI is equivalent to the iFR post-TAVI. Previous studies in the field have also demonstrated identical values of iFR before and after TAVI.^11^ The same study also suggested a 15% classification change of coronary stenosis significance by iFR after TAVI, but this is confounded by the use of a conventional 0.89 cut point and also the distribution of iFR values close to this cut point.^12^ As in our present study, the iFR and fractional flow reserve values were similar pre-TAVI reflecting the inability of adenosine to augment flow in patients with severe AS.

Post-TAVI, resting microvascular resistance increases because one lesion affecting coronary flow has been treated. It does not normalize, however, as there is a residual coronary stenosis that needs to be accommodated. The change in microvascular resistance post-TAVI is independent of the underlying coronary stenosis.

Furthermore, in this study, we compare the increase in resting microvascular resistance post-TAVI to the effect of treating a coronary stenosis in a cohort of patients with a coronary stenosis but no AS. When we do this, it can be seen that stenting coronary stenoses with iFR values <0.74 are able to produce increases in microvascular resistance equivalent to that observed by treating the AS. This is likely to be a conservative estimate because treating a coronary lesion in a patient post-TAVI may not lead to equivalent microvascular change; due to factors such as advanced age, left ventricular hypertrophy, and elevated left-ventricular end-diastolic pressure.^13,14^ As a result, in patients with severe AS, the coronary stenosis may have to be even more severe to achieve similar increases in microvascular resistance as those seen by treating the AS. This would suggest that in patients with AS and coronary disease the predominant lesion is the aortic valve unless the coronary stenosis has an iFR value <0.74.

### Clinical Implications

It is not uncommon for patients with severe AS referred for TAVI to have concomitant CAD.^15^ Both conditions can present with angina or dyspnea on exertion. It can therefore be challenging for clinicians to determine which lesion is predominantly responsible for an individual patient’s presentation. There is currently no clear evidence that PCI before TAVI improves clinical outcomes^16^ but the importance of accurately assessing the functional significance of coronary disease in these patients is becoming increasingly important as TAVI is being offered to younger, lower-risk patients.

Our study suggests that in such patients, coronary physiology can help to clarify the situation. In patients with severe AS and coronary lesions, if the iFR value is >0.74, then it is likely that TAVI will lead to a greater improvement in coronary hemodynamics than PCI—and may therefore be the preferred initial strategy. Conversely, if the iFR value is <0.74, then the coronary stenosis may provide a greater contribution to the patient’s hemodynamic status. In such a situation, the treating clinician may give greater consideration to treating the coronary lesion in addition to the valve.

This iFR value of 0.74 is not designed to be interpreted as a hard cut point to guide PCI or defer TAVI. Rather, it is more intended to provide a framework for clinicians when treating this challenging patient cohort; when it is unclear whether the aortic valve stenosis or coronary stenosis is the major factor in the patient’s presentation. Ultimately, in the absence of robust randomized data in this field, the decision of whether to perform PCI in patients with severe AS scheduled for TAVI must be undertaken on a case by case basis and after the deliberation of the Heart Team. This study suggests iFR may add to these deliberations, along with other factors such as the location of the coronary stenosis, the amount of subtended myocardium, suitability for dual antiplatelet therapy,^17^ the ability to access the coronary ostia post-TAVI and the patient’s symptoms.

Our findings should be considered hypothesis-generating, and the true clinical value of intracoronary physiology in patients with severe AS will only be appreciated when tested in prospective fashion in a clinical trial.

## Limitations

The analysis performed in this study compared the hemodynamic benefit of TAVI with the hemodynamic benefit of PCI. The patients undergoing PCI did not have severe AS, and there were other baseline differences in the groups. These differences are likely to underestimate the true effect of TAVI on coronary flow.^18–20^

Our post-TAVI measurements were all made within the same cath-lab procedure, immediately after the aortic valve had been replaced. This helped to minimize the effect of any potential confounding factors and to truly isolate the effect of the TAVI on coronary hemodynamics. It is possible that there would be further longer-term hemodynamic benefits of TAVI, which would be seen with regression of left ventricular mass and remodeling of the ventricle. We cannot comment on this from our study, but it is the subject of ongoing research. Regardless, the decision to intervene on a coronary stenosis in the context of AS is clinically most relevant before valve treatment, suggesting that the acute effect of TAVI on the microcirculation is most relevant for this analysis and in clinical practice.

We have demonstrated that, in the presence of both AS and CAD, TAVI improves microcirculatory perfusion. However, we cannot tell if (1) concomitant treatment of coronary artery stenosis with percutaneous intervention would have afforded additional benefits; (2) or, if in fact treating CAD would have reduced the benefits of TAVI on microcirculation, as it is conceivable that the negative effects of severe AS could be more prominent in the presence of concomitant CAD.

A larger proportion of patients in the PCI group had more severe coronary lesions with lower iFR values than in the TAVI group. Nevertheless, our patients were representative of a clinical population with severe AS and CAD who were referred for TAVI.

### Conclusions

TAVI improves microcirculatory function regardless of the severity of underlying coronary disease. TAVI for severe AS produces a coronary hemodynamic improvement equivalent to the hemodynamic benefit of stenting coronary stenoses with iFR values <0.74. Future trials of physiology-guided revascularization in severe AS may consider using this value to guide treatment of concomitant CAD.

## Sources of Funding

We are grateful for infrastructural support from the NIHR Biomedical Research Centre based at Imperial College Healthcare NHS Trust and Imperial College London. The views expressed are those of the authors and not necessarily those of the NIHR or the Department of Health and Social Care. C. Cook (MR/M018369/1) and Dr Sen (G1000357) are supported by the Medical Research Council. J.P. Howard is supported by the Wellcome Trust (PS3162_WHCP). Dr Petraco (FS/11/46/28861), Dr Davies (FS/05/006), and Dr Francis (FS 04/079) are supported by the British Heart Foundation.

## Disclosures

Drs Gotberg, Cook and Petraco have conducted teaching sessions supported by Volcano Corporation. Dr Sen has attended and conducted teaching sessions supported by Volcano Corporation, St. Jude Medical, Medtronic, Pfizer, and AstraZeneca; has received research grant support from Philips, AstraZeneca, Medtronic, and Pfizer; and has received speaking honoraria from Pfizer and Volcano-Philips. Drs. Mayet and Davies hold patents pertaining to the instantaneous wave–free ratio technology, which is under license to Volcano Corporation. Dr Davies has served as a consultant for and has received significant research funding from Volcano Corporation. Professor Serruys has received grants and personal fees from Phillips Medtronic and ReCor Medical. The other authors report no conflicts.
